# Trends in socioeconomic inequalities in child undernutrition: Evidence from Nigeria Demographic and Health Survey (2003 – 2013)

**DOI:** 10.1371/journal.pone.0211883

**Published:** 2019-02-07

**Authors:** Blessing J. Akombi, Kingsley E. Agho, Andre M. Renzaho, John J. Hall, Dafna R. Merom

**Affiliations:** 1 School of Social Sciences and Psychology, Western Sydney University, Penrith, New South Wales, Australia; 2 School of Science and Health, Western Sydney University, Penrith, New South Wales, Australia; 3 School of Public Health and Community Medicine, University of New South Wales, Sydney, New South Wales, Australia; The University of Warwick, UNITED KINGDOM

## Abstract

**Objective:**

The aim of this study was to examine the trend in socioeconomic inequalities in child undernutrition in Nigeria.

**Methods:**

The study analysed cross-sectional data from the Nigeria Demographic and Health Survey (NDHS) 2003 to 2013. The outcome variables were stunting, wasting and underweight among children under-five years. The magnitude of child undernutrition in Nigeria was estimated via a concentration index, and the socioeconomic factors contributing to child undernutrition over time were determined using the decomposition method.

**Results:**

The concentration index showed an increase in childhood wasting and underweight in Nigeria over time. The socioeconomic factors contributing to the increase in child undernutrition were: child’s age (0–23 months), maternal education (no education), household wealth index (poorest household), type of residence (rural) and geopolitical zone (North East, North West).

**Conclusions:**

To address child undernutrition, there is a need to improve maternal education and adopt effective social protection policies especially in rural communities in Nigeria.

## Introduction

Child undernutrition is a major public health problem in many low and middle income countries (LMICs). It results from a complex interaction of contextual factors related to community, household, environmental, socioeconomic and cultural influences which has significant health consequences [1]. Undernutrition leads to sub-optimal intellectual and physical development, thus adversely affecting educational performance, economic productivity and reproductive outcomes in adulthood [2].

Globally 45% of deaths among children under-five years are linked to undernutrition [3]. Though the global prevalence of stunting, wasting and underweight has declined from 32.7%, 9% and 25%, respectively, in 2000, to 22.9%, 7.7% and 15%, respectively, in 2016, evidence shows that the burden of child undernutrition is not equally distributed within regions and countries of the world [4]. One-third of all undernourished children live in sub-Saharan Africa (SSA), and Nigeria have reported one of the highest prevalence of undernutrition within SSA. In Nigeria, childhood stunting declined from 42% in 2003 to 36% in 2013, while wasting and underweight increased from 11% and 24%, respectively, in 2003 to 18% and 29%, respectively, in 2013 [5, 6]. This increase in wasting and underweight despite the overall decline in stunting suggests that child undernutrition is still a major public health concern in Nigeria.

Previous studies conducted in Nigeria [7, 8] and other SSA countries [9, 10, 11] have reported socioeconomic factors relating to household income, rural/urban residence, parents’ education and occupation to be associated with child undernutrition. These socioeconomic factors determine the extent of food (in) security [12], access and utilization of health care services [13], as well as exposure to appropriate hygiene and sanitation. Recent studies have also reported that improvements in socioeconomic factors such as female education, level of food security, urbanization, conditional cash transfers and universal health care coverage could significantly reduce childhood undernutrition [14, 15]. However, these studies were limited in scope and did not analyse the trend in socioeconomic inequality as well as examine the contributing factors to this inequality.

This study examined the association between socioeconomic factors and undernutrition. It reports the trend in socioeconomic inequalities as well as examines the contributing factors to this inequality. This study draws attention to different patterns of inequality within vulnerable subpopulations over the period 2003–2013 and contributes to the existing body of evidence on child undernutrition needed to inform policy formulation and intervention strategies to improve child nutrition in Nigeria.

## Methods

### Data sources

The datasets analysed in this study were obtained from 2003 and 2013 Nigeria Demographic and Health Surveys (NDHS). A pooled sample of the two surveys yielded 22217 children aged 0–59 months. The NDHS is a nationally representative survey which collect data on mortality, fertility, family planning and maternal and child health. The survey employs a stratified, multi-stage (cluster), random sampling design. Information on socioeconomic, demographic, environmental, and health characteristics of households was obtained by interviewing women aged 15–49 years, men aged 15–59 years, and collecting the anthropometry of children under-5 years. Detailed survey methodology and sampling techniques are available in the respective NDHS reports [5, 6].

### Dependent variables

The dependent variable analysed in this study was child undernutrition which was categorized into stunting (height-for-age), wasting (weight-for-height) and underweight (weight-for-age). The height-for-age index (HAZ) is an indicator of linear growth impairment and cumulative growth deficits in children, the weight-for-height index (WHZ) indicates recent and severe weight loss, it estimates body mass relative to height or length while the weight-for-age index (WAZ) is a compound index of height-for-age and weight-for-height. The WAZ could reflect both acute weight loss (wasting) and chronic growth failure (stunting), without distinguishing between both forms of undernutrition.

The growth index of children was calculated using growth standards published by the World Health Organization (WHO) in April 2006.These growth standards were generated through data collected in the WHO Multicentre Growth Reference Study [16] and expressed as standard deviation from the Multicentre Growth Reference Study median. Children with index Z-score below minus two standard deviations (-2 SD) from the median of the WHO reference population were used in the study. The 2003 NDHS growth index of children was originally measured using the US National Center for Health Statistics (NCHS) guideline prior to the introduction of the WHO Z-score standard values in 2006. However, for the purpose of assessing trends, the data from the 2003 NDHS were recalculated using the WHO child growth standards adopted in 2006.

### Independent variables

The socioeconomic variables analysed in this study were mother’s age, maternal work status, maternal education, father’s education, household wealth index, geopolitical zone and type of residence. The inclusion of geopolitical zone and type of residence provides insight into the regional clustering of poor–rich disparities in Nigeria. The household wealth index serves as an indicator of the economic status of the household which is consistent with household assets, facilities, income and expenditure measures. The household assets and facilities used in calculating this index include: car, television, motorcycle, electricity, refrigerator, bicycle, radio, type of toilet facility, source of drinking water, and type of building materials used in the place of dwelling. The index was classified into five national-level wealth quintiles via a principal components analysis [17]. The lowest 20% quintile was assigned to the poorest households, the next 20% quintile to the poor households, followed by another 20% quintile for the middle-class households and finally the top 40% quintile for the rich and richest households. Type of residence was grouped into rural and urban settlements. Nigeria has six geopolitical zones which were classified based on ethnic similarity among states having comparable history, cultural beliefs and close territories.

### Statistical analysis

Data analysis was conducted using Stata version 14.0 (StataCorp, College Station, TX, USA). ‘Svy’ commands were used to allow for adjustments in the cluster sampling design. The Taylor series linearization method was used to estimate 95% confidence intervals (CIs) around prevalence estimates. Differences in the socioeconomic variables between the two surveys were expressed as percentages. The significance of association between the surveys was assessed using chi-squared test and a 5% significance level was set. The dependent variables (stunting, wasting, and underweight) were converted into binary variables and multivariable analysis was conducted using the logistic regression model.

### Econometric analysis

Econometric analysis was carried out at two levels. First, the concentration index (CI) with stata command ‘conindex’, which provides point estimates and standard errors [18], was used to measure the extent of inequality associated with wealth and to quantify the magnitude of socioeconomic-related inequality in child undernutrition [19]. The second level analysis used the decomposition method with stata command ‘rdecompose’ to decompose the concentration indices of socioeconomic factors associated with child undernutrition by examining inequality using a set of determinants that vary systematically [20]. The percentage contribution of each factor was reported. The higher the percentage value the greater its contribution to socioeconomic inequality. The mathematical expression for the stata commands “conindex’ and ‘rdecompose’ are explained below.

#### The concentration index (CI)

In order to understand the contributions of individual socioeconomic factors to child undernutrition (underweight, stunting, and wasting), the CI was computed. The CI is calculated as twice the area between the concentration curve and the line of equality (the 45-degree line). O’Donnell [18, 21] described the CI formula as follows: suppose μ_*h*_ is the mean of undernutrition in children under-five, and we have a child undernutrition, *h*, where *h*_*i*_ is child undernutrition for individual *i*. Then if *r*_*i*_ is the fractional rank of individual i in the distribution of household socioeconomic status, the concentration index (CI) is given by:

$$CI=\frac{2*Cov(h_{i},r_{i})}{\mu_{h}}$$ (1)

CI is the mean-adjusted covariance of undernutrition and socioeconomic rank. The CI of child undernutrition can take a value from -1 to +1. If CI is zero, it indicates perfect equality among under-five undernutrition, if the CI is negative, it indicates inequality concentrated among the relatively poor, whereas if CI is positive it indicates that the concentration of under-five undernutrition is higher among the relatively rich.

#### Decomposition of the CI

The distinguishing property of the CI is that it can be decomposed in order to determine both unadjusted and adjusted factors that contribute to inequality in child undernutrition. In the decomposition analysis, child’s age in months was entered into the model as an independent variable alongside mother’s age, maternal working status, maternal education, household wealth index, type of residence and geopolitical zone. This allows for the interpretation of adjusted estimates as a positive or negative percentage contributing of stunting, wasting and underweight. If the vector ***X*** refers to those variables influencing *h*, and we assume that the child undernutrition can be described by a linear regression of the form:

$$h_{i}=\alpha +\beta_{k}X_{ki}+\varepsilon_{i}$$ (2)

then CI can be written as:

$$CI=\sum_{k}(\frac{\beta_{k\;{\bar{X}}_{k}}}{\mu_{h}}){CI}_{k}+\frac{G{CI}_{g}}{\mu_{h}}$$ (3)

where the index *k* refers to the regressors in the equation, *CI*_*k*_ is the concentration index for each of the individual regressors, *β*_*k*_ is the coefficient for each child undernutrition determinant and *X*_*k*_ is the mean value of each individual regressor. *GCI*_*g*_ is the generalized concentration index for the residual from the regression (ε_i_).

The above mathematical expressions refer to the situation where child undernutrition is continuous. However, when underweight, stunting or wasting *h*_*i*_ are binary outcomes, *h*_*i*_ takes on a value of 0 or 1. In this case, a normalization *n* must be applied to the CI (since the bounds would not be -1 and +1). In our analysis we applied the Erregeyers [22] normalization CI_*g*_ = 4μ_*h*_CI = 4μ_*h*_ (1 - μ_*h*_)CI_*n*_ to the CI and its decomposition.

## Results

Table 1 reports the CI of undernutrition among children 0–59 months. The CIs of stunting, wasting and underweight differ statistically and showed a negative value for stunting, wasting and underweight. The negative CIs signify that stunting, wasting and underweight concentrated among children from poor households. A more detailed look at Table 1 show that CI of stunting was greater in 2003 than in 2013, indicating that stunting inequality decreased during this period, while wasting and underweight inequality increased. However, the difference in the CIs for stunting, wasting and underweight between 2003 and 2013 did not differ statistically.

**Table 1:** Concentration indices (CI) of undernutrition among children 0–59 months, NDHS 2003 and 2013.

	Stunting (HAZ<2SD)		Wasting (WHZ<2SD)		Underweight (WAZ<2SD)	
	CI [SE]	*P*	CI [SE]	*P*	CI [SE]	*P*
Year 2003	-0.132 [0.0157]	<0.001	-0.050 [0.0299]	0.0953	-0.134 [0.0229]	<0.001
Year 2013	-0.149 [0.0096]	<0.001	-0.047 [0.0158]	0.0032	-0.125 [0.0133]	<0.001
Diff	-0.017 [0.0185]	0.3564	0.003 [0.0338]	0.9241	0.009 [0.0266]	0.7487

SE: standard error. Diff: difference in CI between 2003 and 2013. Note: If CI is negative = inequality is concentrated among the relatively poor; If CI is positive = inequality is concentrated among the relatively rich; If CI is Zero = no inequality in child undernutrition.

Table 2 shows the prevalence of undernutrition among children 0–59 months by wealth index and reports its trend between 2003 and 2013. The overall prevalence of childhood stunting by wealth index significantly decreased while wasting and underweight significantly increased between 2003 and 2013. A detailed intra-quintile analysis also showed that stunting increased within each wealth index quintile while wasting and underweight decreased within each quintile.

**Table 2:** Prevalence of undernutrition among children 0–59 months by wealth index, NDHS 2003 and 2013.

	Poorest	Poorer	Middle	Richer	Richest	Overall
**Stunting (HAZ < 2SD)**						
Year 2003	55.9 [51.5, 60.2]	52.0 [47.1, 56.9]	49.2 [44.6, 53.9]	38.4 [33.2, 43.8]	24.8 [20.1, 30.3]	45.0 [41.9, 48.1]
Year 2013	47.0 [43.8, 50.1]	46.5 [44.0, 49.1]	41.9 [39.3, 44.5]	31.2 [29.1, 33.4]	19.6 [17.5, 21.8]	36.8 [35.2, 38.4]
Diff-1 [SE]	- 8.9 [0.027]	- 5.5 [0.029]	- 7.3 [0.027]	- 7.2 [0.029]	- 5.3 [0.028]	-8.2 [0.019]
**Wasting (WHZ < 2SD)**						
Year 2003	14.4 [12.1, 17.0]	14.5 [11.4, 18.3]	11.0 [8.7, 13.8]	14.6 [11.6, 18.1]	10.0 [7.3, 13.6]	13.0 [11.6, 14.5]
Year 2013	20.2 [18.2, 22.3]	19.0 [17.1, 21.0]	18.7 [16.9, 20.6]	19.3 [17.1, 21.6]	14.2 [12.2, 16.4]	18.4 [17.1, 19.7]
Diff-2 [SE]	5.8 [0.016]	4.4 [0.020]	7.7 [0.016]	4.7 [0.020]	4.1 [0.019]	5.4 [0.009]
**Underweight (WAZ < 2SD)**						
Year 2003	35.3 [31.4,39.3]	30.4 [25.6,35.6]	26.8 [23.0,31.0]	24.0 [20.2,28.2]	16.1 [11.8,21.6]	27.2 [24.6,29.8]
Year 2013	35.7 [32.9, 38.5]	35.6 [33.0, 38.3]	31.1 [28.7, 33.6]	26.0 [23.4, 28.7]	17.2 [14.7, 20.0]	28.9 [27.2, 30.6]
Diff-3 [SE]	0.4 [0.025]	5.2 [0.029]	4.2 [0.024]	2.0 [0.025]	1.1 [0.029]	1.7 [0.016]

SD = standard deviation; SE = standard error; Diff-1, Diff-2, Diff-3 = the difference in prevalence between 2003 and 2013 of under-five children who were stunted, wasted and underweight respectively.

Table 3 presents the socioeconomic factors associated with child undernutrition among children 0–59 months. The significant factors associated with childhood stunting include; child’s age (older child age), mother’s age (≤ 24 years), sex of child (male child), maternal education (no education), father’s education (no education), household wealth index (poorest household), type of residence (rural) and geopolitical zone (North West, North East and North central). The significant factors associated with wasting and underweight were child’s age (older child age), sex of child (male child), maternal education (no education), father’s education (no education), and geopolitical zone (North West, North East and North central).

**Table 3:** Socioeconomic factors associated with undernutrition among children 0–59 months, NDHS 2003 and 2013.

Characteristics	Stunting		Wasting		Underweight	
	OR [95% Cl]	*p-value*	OR [95% Cl]	*p-value*	OR [95% Cl]	*p-value*
Year 2003	1		1		1	
Year 2013	0.71 [0.65, 0.76]	<0.001	1.51 [1.34, 1.69]	<0.001	1.04 [0.95, 1.14]	0.431
Child's age (Months)	1.02 [1.02, 1.02]	<0.001	0.97 [0.97, 0.98]	<0.001	1.00 [1.00, 1.00]	<0.001
**Mother's age**						
≤ 24 years	1		1		1	
25–34 years	0.87 [0.81, 0.93]	<0.001	1.07 [0.99, 1.17]	0.098	0.95 [0.88, 1.02]	0.145
35 and above	0.83 [0.77, 0.90]	<0.001	1.02 [0.92, 1.13]	0.715	0.89 [0.82, 0.98]	0.011
**Sex of child**						
Male	1		1		1	
Female	0.79 [0.75, 0.84]	<0.001	0.86 [0.81, 0.92]	<0.001	0.82 [0.78, 0.87]	<0.001
**Maternal working status**						
Non-working	1		1		1	
Working (past 12 months)	0.97 [0.91, 1.03]	0.251	0.97 [0.90, 1.05]	0.456	1.00 [0.94, 1.07]	0.843
**Maternal education**						
No education	1		1		1	
Primary	0.94 [0.87, 1.03]	0.179	0.95 [0.85, 1.06]	0.385	0.93 [0.85, 1.02]	0.125
Secondary and above	0.72 [0.65, 0.79]	<0.001	0.86 [0.75, 0.97]	0.016	0.75 [0.67, 0.83]	<0.001
**Father’s education**						
No education	1		1		1	
Primary	1.03 [0.94, 1.11]	0.561	0.95 [0.86, 1.06]	0.384	1.00 [0.92, 1.09]	0.977
Secondary and above	0.86 [0.79, 0.94]	<0.001	0.86 [0.77, 0.97]	0.010	0.85 [0.77, 0.93]	<0.001
**Household wealth index**						
Poorest	1		1		1	
Poorer	0.98 [0.89, 1.07]	0.588	0.98 [0.87, 1.10]	0.754	0.97 [0.88, 1.06]	0.458
Middle	0.92 [0.84, 1.00]	0.052	1.02 [0.90, 1.15]	0.772	0.90 [0.82, 0.99]	0.034
Richer	0.74 [0.67, 0.82]	<0.001	1.09 [0.96, 1.24]	0.198	0.79 [0.71, 0.88]	<0.001
Richest	0.53 [0.47, 0.59]	<0.001	0.92 [0.79, 1.08]	0.333	0.66 [0.57, 0.75]	<0.001
**Type of residence**						
Urban	1		1		1	
Rural	1.16 [1.06, 1.26]	0.001	0.89 [0.79, 1.00]	0.056	0.95 [0.86, 1.05]	0.276
**Geopolitical Zone**						
North central	1		1		1	
North East	1.43 [1.27, 1.60]	<0.001	1.69 [1.44, 2.00]	<0.001	1.78 [1.56, 2.02]	<0.001
North West	2.61 [2.33, 2.92]	<0.001	2.38 [2.03, 2.78]	<0.001	3.30 [2.91, 3.74]	<0.001
South East	0.52 [0.44, 0.61]	<0.001	0.92 [0.74, 1.14]	0.432	0.59 [0.49, 0.72]	<0.001
South West	0.65 [0.56, 0.75]	<0.001	1.06 [0.87, 1.30]	0.543	0.75 [0.63, 0.89]	0.001
South South	0.86 [0.75, 0.98]	0.030	0.96 [0.78, 1.17]	0.665	0.88 [0.75, 1.04]	0.129

Table 4 shows the adjusted estimates for the percentage contribution of socioeconomic factors to overall CIs of stunting, wasting and underweight in children under-five years in 2003 and 2013.

**Table 4:** Adjusted estimates for percentage contribution of socioeconomic factors to child undernutrition in 2003 and 2013.

Characteristics	Stunting (%)		Wasting (%)		Underweight (%)	
	2003	2013	2003	2013	2003	2013
**Child's age**						
0–23 months	190.2	352.1	-489.1	201.8	7.2	19.9
24–59 months	Ref	Ref	Ref	Ref	Ref	Ref
**Mother's age**						
15–24 years	48.0	70.9	2.7	79.7	9.8	-37.1
25–34 years	-133.3	-140.9	-7.9	-16.9	-54.7	19.1
35 and above	Ref	Ref	Ref	Ref	Ref	Ref
**Maternal working status**						
Working (past 12 months)	61.0	106.3	183.3	-36.7	35.8	25.8
Non-working	Ref	Ref	Ref	Ref	Ref	Ref
**Maternal education**						
No education	70.5	118.8	123.3	137.4	32.0	57.9
Primary	-322.7	-554.4	-180.6	-75.7	-82.2	-150.7
Secondary and above	Ref	Ref	Ref	Ref	Ref	Ref
**Household wealth index**						
Poorest	186.2	264.1	-120.1	52.5	46.8	58.7
Poorer	-107.0	-187.6	-92.7	47.9	1.6	-33.7
Middle	-152.6	-409.2	214.3	23.4	-26.3	-97.6
Richer	-311.2	-436.3	-42.7	8.4	-80.8	-100.6
Richest	Ref	Ref	Ref	Ref	Ref	Ref
**Type of residence**						
Urban	-140.5	-239.2	-35.4	18.0	-27.4	-49.1
Rural	Ref	Ref	Ref	Ref	Ref	Ref
**Geopolitical Zone**						
North central	278.9	323.9	249.2	-227.5	110.0	150.5
North East	525.7	1127.5	768.7	266.7	176.7	293.5
North West	533.9	769.4	-160.6	43.7	125.1	246.8
South East	-103.8	-311.6	1.0	-230.4	-35.0	-65.7
South West	-523.3	-753.8	-313.4	-192.3	-138.6	-237.7
South South	Ref	Ref	Ref	Ref	Ref	Ref
**Total**	100	100	100	100	100	100

Note: A positive contribution indicates that the respective variable is increasing socioeconomic inequality. The higher the percentage value the greater its contribution to socioeconomic inequality.

### Stunting

Trends analysis showed the main factors contributing to socioeconomic inequality in child stunting were: child’s age (0–23 months), mother’s age (>24 years), maternal working status (working mothers), maternal education (no education), household wealth index (poorest households), and geopolitical zone (North central, North East and North West).

### Wasting

The main factors which reported an increase in contribution to socioeconomic inequality in wasting from 2003 to 2013 were: child’s age (0–23 months), mother’s age (>24 years), maternal education (no education), type of residence (urban), household wealth index (poorest households), and geopolitical zone (North West).

### Underweight

The main factors which contributed to an increase in socioeconomic inequality in underweight from 2003 to 2013 were: child’s age (0–23 months), maternal education (no education), household wealth index (poorest households), and geopolitical zone (North central, North East and North West).

## Discussion

This study examines the trend in socioeconomic inequalities associated with child undernutrition in Nigeria. The factors that showed the largest contribution to socioeconomic inequalities in child undernutrition over time were child’s age (0–23 months), maternal education (no education), mother’s age (≤ 24 years), household wealth index (poorest household), type of residence (rural) and geopolitical zone (North East and North West).

This study shows that socioeconomic factors greatly influence nutrition in Nigeria. The disparity in nutrition status within subpopulations disproportionately affects the poor and uneducated, especially in rural areas. The observed increase in undernutrition among poor households widens the poor-rich divide and reflects further deterioration in the living conditions of families over the years in Nigeria. This trend in disparity could be due to changes in income, education, lifestyle, and other factors that directly or indirectly affect food security [23, 24]. The Nigerian economy was reported as one of the fastest growing economies in the world [25], however, in recent times the pace of growth in Nigeria has slowed [26, 27]. Recent economic statistics reveal a contrast between rapid economic growth and welfare improvements for much of the population [21]. Poverty reduction and job creation have not kept pace with population growth, implying social distress for an increasing number of households, particularly in rural areas. With higher energy prices, poor weather conditions, and growing security challenges in some parts of the Northern geopolitical region, the number of poor households living below the poverty line has grown measurably [25, 26, 27].

Findings from this study are consistent with previous studies conducted on the disparities in nutrition health outcomes between the poor and the rich which reported that poorer children are at a higher risk of undernutrition [28, 29, 30]. The socioeconomic status of households affects their access to quality food, supply of clean water, improved sanitation facilities, and basic healthcare facilities [31–34]. Previous studies have shown that the lack of access to a stable food supply due to financial constraints has an adverse effect on child growth [35, 36, 37]. The unavailability of safe drinking water and proper environmental sanitation experienced by poor households especially in rural areas is a major cause of diarrhoea, which has been reported to be a determinant of undernutrition [15, 38, 39]. The role of the health sector is important in mitigating the impact of poverty on undernutrition by providing basic health amenities such as clean drinking water, appropriate waste disposal systems and access to health care facilities.

The regional clustering of socioeconomic inequalities falls disproportionately on the northern geopolitical zone of Nigeria, which has reported a higher proportion of uneducated mothers and poorer health care system compared to the southern zone [7, 30], a disparity that increased from 2003 to 2013. The increasing trend in child undernutrition in northern Nigeria is compounded by the overall status of women in the region, where pregnancies at a young age (15–19 years) are very high and women’s education levels are very low [30]. Thus improving literacy among females should be prioritised in order to address this inequality. The role of mothers’ education has an enormous impact on undernutrition outcomes due to its influence on health-related decisions and the allocation of resources for food within the household [13]. Higher maternal education translates into greater health care utilization, including formal antenatal and postnatal visits. Studies show that adequate antenatal and postnatal care could effectively enhance child growth trajectories [40, 41, 42], and improve maternal health-related parenting practices such as breastfeeding and complementary feeding [43, 44, 45], as well as initiate positive behavioural changes that could translate to better child growth outcomes [46]. The finding from our study is consistent with the result from a cross-sectional study conducted in Ghana using the multiple indicator cluster survey (MICS), which reported an inverse relationship between maternal education and undernutrition [47].

In this study, child’s age was reported as one of the largest contributors to socioeconomic inequalities to undernutrition in children under-five years. Results also show an increase in undernutrition among children aged 0–23 months. Adequate nutrition at this period of growth is crucial to proper cognitive and physical development of the child. The World Health Organization (WHO) recommends exclusive breastfeeding for infants in the first 6 months from birth to achieve maximal physical growth, mental development and overall health [48]. Thereafter, infants should be given appropriate complementary foods that are safe and nutritionally adequate for their evolving nutritional and growth needs without ceasing breastfeeding for up to 24 months or beyond [48]. There is also a global agreement on a critical window—from conception through the first 2 years of life (0–23 months)—within which 70% of growth deficit occurs [49]. This deficit in growth may be due to poor child feeding practices, such as low uptake of exclusive breastfeeding and prolonged duration of breastfeeding without the timely introduction of adequate complementary feeding [48, 49], which may result from inadequate breastfeeding knowledge by mothers [50], lack of support from family and society or lack of guidance and encouragement from health care professionals [50, 51, 52]. To address this increase in undernutrition among children aged 0–23 months, community-based educational programs on appropriate child feeding practices targeting uneducated mothers in rural areas need to be implemented.

Another important finding of this study is the rural-urban clustering of undernutrition. The rural areas reported an increase in the number of stunted and underweight children compared to the urban areas. Studies on socioeconomic inequalities in health have shown evidence of child health differences across rural and urban settings [30, 53]; however some other studies have revealed that urban-rural differentials in child health would be abolished once the socioeconomic variables of these households were controlled [54]. The urban-rural differentials in child undernutrition are accounted for by differences in the distribution of socioeconomic conditions, with the rural areas harbouring pockets of severe poverty, illiteracy, unemployment and exhibiting substantial concentrations of ill-health among the poor [53, 55]. An increase in undernutrition rate within the rural areas indicates a worsening in the health conditions and lack of access to modern health care systems, thus increasing the urban-rural gaps in health. This finding is consistent with results from a cross-sectional study carried out in the Democratic Republic of Congo (DRC), which found that the rate of child stunting was significantly higher in rural areas compared to urban areas [55].

This study had some limitations. Firstly, due to the cross-sectional nature of the study design, it is not possible to establish a causal relationship between the socioeconomic variables and undernutrition. Secondly, the classification of localities into urban and rural areas might be problematic as large cities have heterogeneous areas with regard to these variables, and data to identify such heterogeneity are not available. Thirdly, there was no direct measure for household income. The proxy measures and the construction of an index might have affected the impact of these variables on the dependent variable. Finally, due to the large difference in sample size between 2003 and 2013 NDHS, trend analyses may reflect power differences across survey years, however both surveys where weighted to represent the Nigerian population. Despite these limitations, this study also had some merits. First, data used in this study were from population-based surveys with large sample size and good response rates (> 90). Second, this study applied appropriate statistical adjustments to data obtained from two nationally representative surveys and was able to identify the trends in socioeconomic inequalities in the pooled sample. Finally, this study is useful in identifying the major socioeconomic factors contributing to the increase in child undernutrition in Nigeria to assist in proper public health planning.

### Policy implications

Based on the results from this study, critical policy insights can be deduced. These insights will enable policy makers and public health researchers to develop effective nutrition and education interventions that involve community adapted strategies targeted at improving maternal education by promoting female child education as well as bridging the rural–urban development gap through the provision of accessible health care facilities, portable drinking water and adequate waste disposal systems. Poverty alleviation and social protection programmes such as cash transfers, in-kind transfers, unemployment benefits, child support, national health insurance, job-creation schemes and agricultural insurance aimed at the poorest subpopulations are also needed to improve food security and achieve equitable outcomes.

## Conclusion

Findings from this study show that socioeconomic inequalities are prevalent and have increased in rural areas as well as among uneducated mothers and poor households especially in the Northern geopolitical zones of Nigeria. Therefore, efforts to tackle these inequalities should target the reduction of rural–urban differences in development and alleviate poverty through public health programmes and social protection policies that empower households (especially the mothers) and improve access to food, education and resources; these would reduce child undernutrition in Nigeria, thus setting the country on the path to achieving the WHO global nutrition target by 2025.

## Supporting information

S1 Table STROBE checklist. (DOCX)
